# Numerical Investigation of the Impact of ITO, AlInN, Plasmonic GaN and Top Gold Metalization on Semipolar Green EELs

**DOI:** 10.3390/ma13061444

**Published:** 2020-03-22

**Authors:** Maciej Kuc, Łukasz Piskorski, Maciej Dems, Michał Wasiak, Adam K. Sokół, Robert P. Sarzała, Tomasz Czyszanowski

**Affiliations:** Institute of Physics, Lodz University of Technology, Lodz, Poland

**Keywords:** edge-emitting lasers, semiconductor devices, InGaN/GaN, numerical simulations

## Abstract

In this paper, we present the results of a computational analysis of continuous-wave (CW) room-temperature (RT) semipolar InGaN/GaN edge-emitting lasers (EELs) operating in the green spectral region. In our calculations, we focused on the most promising materials and design solutions for the cladding layers, in terms of enhancing optical mode confinement. The structural modifications included optimization of top gold metalization, partial replacement of p-type GaN cladding layers with ITO and introducing low refractive index lattice-matched AlInN or plasmonic GaN regions. Based on our numerical findings, we show that by employing new material modifications to green EELs operating at around 540 nm it is possible to decrease their CW RT threshold current densities from over 11 kA/cm^2^ to less than 7 kA/cm^2^.

## 1. Introduction

Semiconductor edge-emitting lasers (EELs) based on III-N materials that emit green light at room-temperature (RT) in the continuous-wave (CW) regime suffer from considerably higher current densities at the threshold of laser operation than similar EELs which emit blue light [1]. It is mainly caused by the low differential gain and the small transition matrix element.

There is also an another factor being equally important that relates to the effective confinement of an optical mode in the cladding. Typically, AlGaN layers are being used for this purpose. However, these layers cause unfavorable effects. Aluminum-rich layers may easily decompose under normal environmental conditions, which leads to the laser degradation and its failure. Additionally, AlGaN layers not only have to be grown at a high temperatures, which deteriorates the optical and structural properties of the multiple quantum wells (MQW) in the active region, but also have poor electrical conductivity, which increases the total electrical resistance of the device. On the other hand, removing AlGaN layers reduces the optical mode confinement in the active region, which leads to higher threshold currents. This unfavorable effect can be reduced by increasing either the amount of indium in the InGaN waveguide layers or their thickness. However, such approaches are strongly limited in the case of semipolar oriented laser diodes, due to the high strain induced by indium [2].

As can be seen from the above-mentioned text, new solutions to increase the mode confinement in these structures are pursued. Currently, the most attractive approaches, already proven for blue EELs, involve the use of ITO (indium-tin-oxide) as a partial replacement for the p-GaN in EEL ITO p-claddings [3], using lattice-matched n-AlInN in AlInN EELs [4] or incorporating highly doped (plasmonic) n-GaN as a part of the n-claddings or substrate in GaN^++^ EELs [5]. However, according to [6], it is sufficient to increase the thickness of the InGaN waveguides in blue EELs to achieve efficient confinement of the optical mode within its active region.

In this paper, we provide extensive optimization of the construction of green light emitting EELs with CW RT threshold characteristics. Our analysis employs a self-consistent thermal-electrical-optical model, taking into account the influence of ITO embedded on p-GaN, plasmonic GaN and AlInN integrated with n-GaN. The impact of top gold metalization on CW RT threshold operation is also considered. The modeled structures are compared and optimized in respect to the threshold current density and the optical confinement factor for the laser active region (henceforth simply referred to as confinement factor). Despite the fact that so far there have been particular articles demonstrating edge-emitting lasers using each of the materials as a cladding and/or contact layer, to the best of our knowledge there has not yet been an analysis that would show in a methodical way and compare the impact of ITO, plasmonic GaN and AlInN layers on the operation of CW EELs at RT.

## 2. The Model

**Figure 1:**
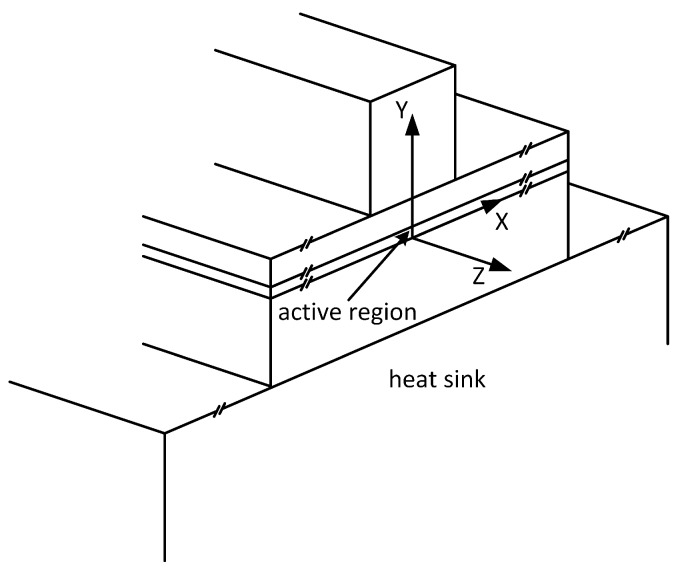
The coordinate system applied within the analysis.

We use a self-consistent model, combining thermal, electrical, optical, and gain submodels to simulate EEL operation. Below, the above-mentioned submodels will be shortly described together with important interactions between them. The coordinate system applied within the calculations is shown in Figure 1.

### 2.1. The Electrical Model

The electrical model of the laser is based on the Laplace equation applied to all layers of the laser structure

(1) ∇[σ∇V]=0,

where σ stands for the electrical conductivity and *V* is the potential distribution. Generation and recombination phenomena within the active region are usually a source of the non-zero right-hand side of Equation (1) (known as the Poisson equation in this case). They are, however, taken symbolically into account in the model with the aid of the effective conductivity $\sigma_{\mathrm{pn}}$ of the active-region material. Its value is determined using the classical diode equation:

(2) $$\sigma_{\mathrm{pn}}\left(x\right)=\frac{\beta_{\mathrm{pn}}j_{\mathrm{pn}}\left(x\right)d_{A}}{\ln\left[\frac{j_{\mathrm{pn}}\left(x\right)}{j_{s}}+1\right]},$$

where $j_{\mathrm{pn}}$ is the p-n junction current density, and $d_{A}$ stands for the total thickness of the active region. Derivation of Equation (2) includes all the above-mentioned phenomena in a natural way: its empirical parameters $\beta_{\mathrm{pn}}$ and $j_{s}$ are dependent on the rates of carrier generation and recombination within the active region.

To obtain potential profile for the whole laser structure, it should be matched (using the self-consistent approach) with the aid of boundary conditions at all boundaries between the layers. Then the current density distribution may be found from the Ohm’s law:

(3) j=−σ∇V.

Afterwards carrier density profile $n_{A}\left(x\right)$ within the active layer may be determined from the threshold diffusion equation

(4) $$D\frac{d^{2}n_{A}\left(x\right)}{dx^{2}}-\left[An_{A}\left(x\right)+Bn_{A}^{2}\left(x\right)+Cn_{A}^{3}\left(x\right)\right]+\frac{j_{\mathrm{pn}}\left(x\right)}{ed_{A}}=0,$$

where *D* stands for the diffusion coefficient, *A* is the monomolecular recombination coefficient, *B* is the bimolecular recombination coefficient, *C* is the Auger recombination coefficient, and *e* is the elementary charge. Equation (4), as well as Equation (1), are solved using the finite element method (FEM).

### 2.2. The Thermal Model

In the thermal model of the laser, the heat transfer equation

(5) −∇[k∇T]=Q,

is solved for the whole structure. In Equation (5), *k* stands for the thermal conductivity of the medium and *Q* is the heat source density. Equation (5) is solved using FEM. We assume that the bottom of the copper heat sink is kept at a constant temperature of 300 K, and the other sides of the laser are thermally isolated.

### 2.3. The Optical Model

The optical phenomena in the laser are modeled with the use of the effective-index method [7]. The optical field Φ(x,y) is expressed as:

(6) $$\Phi \left(x,y\right)=\Phi_{y}\left(y\right)\Phi_{\mathrm{xy}}\left(x,y\right).$$

Then the two-dimensional optical problem is reduced to two nearly one-dimensional problems described by the equations:

(7) $$\frac{\partial^{2}\Phi }{\partial x^{2}}+\frac{\partial^{2}\Phi }{\partial y^{2}}+\left[n_{R}^{2}\left(x,y\right)k^{2}-\beta^{2}\right]\Phi =0,$$

and

(8) $$\frac{\partial^{2}\Phi_{x}}{\partial x^{2}}+\left[n_{\mathrm{eff}}^{2}\left(x\right)k^{2}-\beta^{2}\right]\Phi_{x}=0,$$

which are solved using standard methods. In the above equations, $n_{R}$ and $n_{\mathrm{eff}}$ are the refractive index and the effective index of refraction respectively, *k* is the wave vector and β stands for the propagation constant. The lasing threshold is found from the condition of real propagation constant, which means that for the mode under consideration an imaginary part of its propagation constant vanishes at threshold.

### 2.4. The Gain Model

The optical gain spectra *g* may be then determined from the following relation [8]:

(9) $$g\left(E\right)=\sum_{m}\int_{-\infty }^{\infty }g_{m}\left(\epsilon \right)\Lambda \left(E-\epsilon \right)d\epsilon ,$$

where *E* stands for the energy of the photon. The summation is carried out over all available numbers *m* of level pairs and the integration is carried out over the whole energy ϵ range. Parameter $g_{m}$ from Equation (9) can be obtained from the following equation:

(10) $$g_{m}\left(\hbar \omega \right)=\frac{\pi e^{2}\hbar }{n_{R}m_{0}^{2}c\epsilon_{0}\epsilon }\rho^{2}D\left(\epsilon \right){\left|M\right|}^{2}\left\{f_{c}\left[E_{e}\left(m,\epsilon \right)\right]-f_{v}\left[E_{h}\left(m,\epsilon \right)\right]\right\},$$

where *ℏ* is the Dirac constant, $m_{0}$ is the electron rest mass, *c* is the speed of light in vacuum, $\epsilon_{0}$ is the electric constant, $\rho_{r}^{2}D$ is the two-dimensional reduced density of states, ${\left|M\right|}^{2}$ is the momentum matrix element, $f_{c}$ and $f_{v}$ are the Fermi-Dirac functions determined for electrons in the conduction band and for holes in the valence band, respectively, $E_{e}$ and $E_{h}$ are energies of the recombining electron and hole, respectively. The broadening function Λ depends on lifetime of the stimulated emission $\tau_{\mathrm{st}}$:

(11) $$\Lambda \left(E-\epsilon \right)=\frac{1}{\pi \tau_{\mathrm{st}}\left[\frac{{\left(E-\epsilon \right)}^{2}}{\hbar^{2}}+\frac{1}{\tau_{\mathrm{st}}^{2}}\right]}.$$

In the calculation of the luminescence and optical gain spectra, the classical Fermi’s golden rule [9] and the parabolic band-gap approximation are assumed.

### 2.5. Interactions between Individual Physical Phenomena

The above well-conducted theoretical approach allows an integration of various physical phenomena taking place within a EEL and crucial for its RT CW operation with the aid of the self-consistent approach. In the calculations, all the most important interactions between individual physical phenomena are taken into account including:

- temperature and carrier-concentration dependencies of electrical conductivities,
- temperature dependence of recombination coefficients,
- temperature dependence of thermal conductivities,
- temperature dependence of energy gaps,
- temperature, wavelength and carrier-concentration dependencies of refractive indices and absorption coefficients,
- the effect of the spontaneous and piezoelectric polarizations on the band structure of the active region.

More details of a self-consistent thermal-electrical-optical-gain model for EELs operating at CW RT at the threshold can be found in [10,11,12]. Moreover, in [12] we present the method used to determine the energy band diagram of the MQW structure in the growth direction, which is later used in gain calculations.

Our simulations are performed in 2D, in the plane XY (see Figure 1). Hence, we assume homogeneity of the structure and results in the direction of the light propagation (along Z-axis). This simplification makes the computation reasonably fast, but leads for example to overestimation of the temperature. We also assume that the QW potential does not depend on the current, because at the threshold and above the concentration of carriers in the active region is approximately constant. As a result, the effect of screening of the electric fields in QWs is considered independent from the current if the current is high enough.

## 3. Modeled Structures and Parameters

In this section, we present the analysis of different designs of green EEL, with particular attention to the influence of cladding layers on the threshold parameters. First, we describe the parameters of a reference structure optimized with respect to the lowest threshold current density. Next, we describe the influence of the construction parameters of the cladding layers, composed of ITO, plasmonic GaN and n-AlInN with top gold metalization, on the green EEL designs.

**Figure 2:**
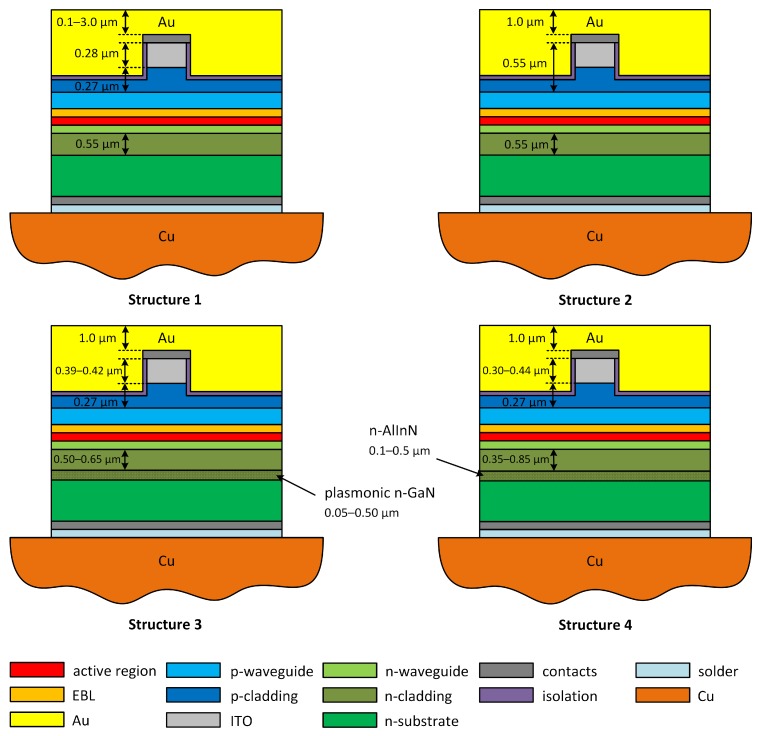
Schematics of considered EEL design. Structure 1: structure with various thicknesses of top gold metalization; Structure 2: structure with various ITO layer thicknesses; Structure 3: structure with GaN:Ge plasmonic layers; and Structure 4: structure with n-AlInN layers.

In this paper, we consider a 2-μm-wide ridge-waveguide EEL emitting in the green range. The construction details are presented in Table 1 and Figure 2. In our calculations, we assumed the width of the EELs to be equal to 500 μm [13]. The exact number of nodes depends on the modeled structure, but due to the fact that all structures are similar, the number of nodes is about 90,000 for each laser. This value corresponds, however, to the half-model meshes, which were used in calculations as the modeled lasers are symmetrical with respect to the Y-axis.

In accordance with [13], we assumed the thickness of the bulk-GaN substrates to be equal to 50 μm. The EELs were mounted p-up on copper blocks using 1-μm-thick PbSn solders. The electrical resistances of the p-type Ti/Au and n-type Al/Au contacts were assumed to be $5\times 10^{-4}\;\Omega \cdot {\mathrm{cm}}^{2}$ and $5\times 10^{-5}\;\Omega \cdot {\mathrm{cm}}^{2}$, respectively. Based on [14], the contact resistances were independent of the calculated current densities. Such high resistance in the p-contact layer is typical for III-N-based EELs [14].

Table 2 presents the thermal and electrical conductivities of the (Al,In)GaN materials [10] and metals [11] used in the calculations. The impact of size-effects and impurities on the thermal conductivity of GaN, as reported recently in [15] and supported in [10], was also taken into account in our simulations.

The refractive indices of (In)GaN given in Table 3 were calculated using the formulas presented in [16]. The refractive index of Al_0.2_Ga_0.8_N was taken from [17,18]. The refractive index and absorption of Al_0.83_In_0.17_N (lattice-matched to GaN) was taken from [19]. Based on [20,21] and the Drude model [12], we assumed the absorption for the n-type (In)GaN materials to be equal to 10 cm^−1^. Based on [20,22], we assumed absorption for all p-type (Al,In)GaN layers to be equal to 100 cm^−1^. The impact of temperature on absorption was not taken into consideration in our simulations.

The parameters of the gain model have been described in [12], with the exception of the matrix element equal to 6 m_0_·eV which was calculated from the Kane model.

**Figure 3:**
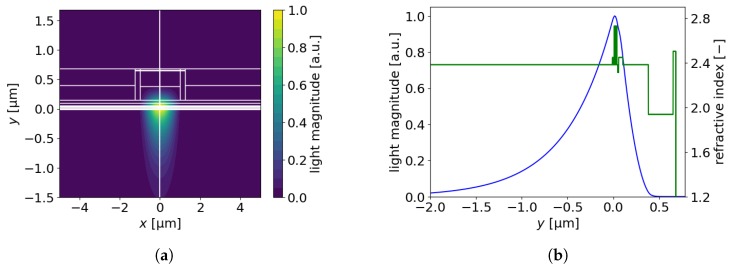
Distributions of light magnitude (**a**) in the xy-plane and (**b**) along the *y*-axis (x=0 μm), with corresponding refractive index distribution.

For parameters $\beta_{\mathrm{pn}}=8.2\;V^{-1}$ and $j_{s}=1.0\times 10^{-7}\;\mathrm{kA}/{\mathrm{cm}}^{2}$ the threshold current density with a maximum of 11.31 kA/cm^2^ calculated for the reference structure corresponds well with [1]. The threshold current was 368 mA and the optical confinement factor, calculated as the ratio of light intensity integrated over the active region to light intensity integrated over the whole computational window along the y-axis, was 7%. The optical mode and the refractive index across the EEL layers are presented in Figure 3a,b. In contrast to the p-type layers, the mode is weakly confined by the n-type layers and leaks into the substrate. The confinement factors in the p-type and n-type layers were 20% and 73%, respectively.

### 3.1. Top Metalization

**Figure 4:**
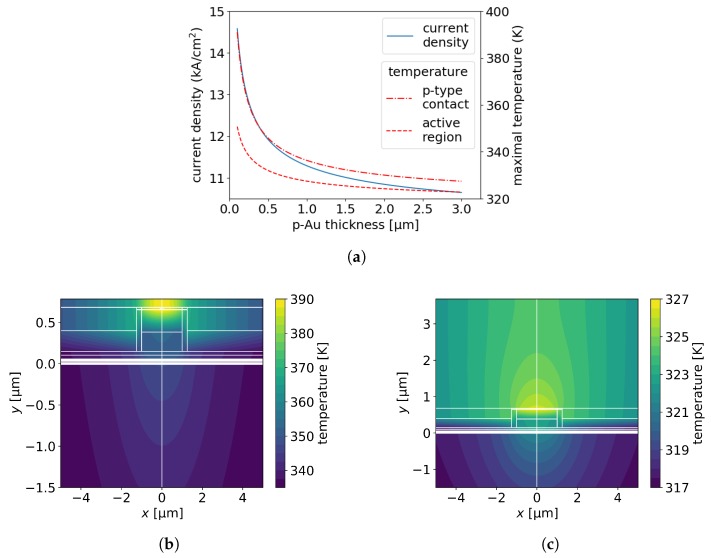
(**a**) Threshold current density and maximal temperature in the p-contact and active region of the modeled EEL as a function of the thickness of top gold metalization. Temperature distributions in the xy-plane for EELs with (**b**) 0.1-μm-thick and (**c**) 3-μm-thick top gold metalization.

As we have reported previously [11], the thermal performance of a p-up mounted EEL can be tuned by changing its top gold metalization. Increasing the metalization thickness decreases the maximal temperature in the active region, which in turn lowers the threshold current density (Figure 4a–c).

Increasing the thickness of the p-Au contact in modeled device from 0.1 μm to 3.0 μm leads to a decrease in the thermal impedance from 10.2 K/W to 7.8 K/W. This, in turn, decreases the threshold current density from 14.6 kA/cm^2^ to 10.7 kA/cm^2^. Moreover, increasing the p-Au contact thickness reduces the difference between the maximal temperatures in the p-contact and active regions, from 40 K (391 K–351 K) to 4 K (327 K–323 K). The confinement factor does not depend on the thickness of the top metalization, and is equal to 7% as for the reference structure. In the next three sections we use p-Au contact with thickness of 1.0 μm, for which the threshold current density is only 5% higher than the one for three times thicker p-Au layer.

### 3.2. EEL ITO

In the EEL reported in [6], the 0.55-μm-thick p-cladding consisted of a 0.3-μm-thick p-GaN layer followed by 0.25-μm-thick ITO layer. The p-GaN layer was located between the ITO layer and the InGaN p-waveguide. The p-GaN layer was thus used as a spacer to distance the ITO layer from the active region. In our simulations, the total thickness of the ITO and GaN p-cladding was set at 0.55 μm and each change in ITO thickness was followed by a corresponding modification to the p-GaN thickness.

**Figure 5:**
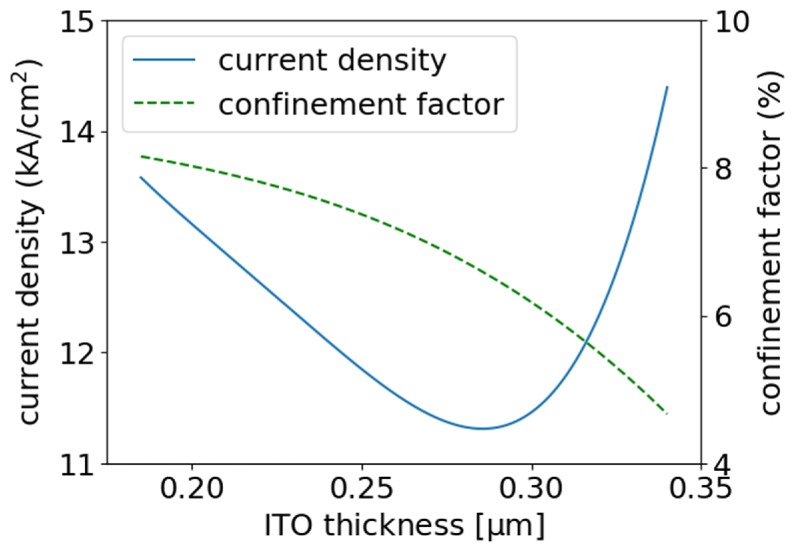
Threshold current densities and confinement factors calculated for the modeled EELs as a function of ITO thickness in ITO/GaN p-claddings.

We investigated the impact of ITO layer thickness on the threshold current density of the modeled laser. The lowest threshold current density, of 11.31 kA/cm^2^, was calculated for a EEL with p-cladding composed of 0.28-μm-thick ITO and 0.27-μm-thick p-GaN layers (Figure 5). The increase in ITO thickness results in pushing the optical mode from the active region towards the n-type GaN cladding, which causes a decrease in the confinement factor and, consequently, an effective drop in gain. This leads to an increase in threshold current. On the other hand, the decrease in ITO thickness results in the loss of optical confinement from the p-side, which enhances the lasing mode penetration into the top Au metalization and causes a significant increase in absorption (losses), which in turn also leads to an increase in threshold current, although the confinement factor for the active region increases, as seen in Figure 5. In the extreme case, for the EEL without the ITO layer, the lasing threshold would not be achieved.

In the next stage of the study, this EEL (referred to as the reference EEL), was used for further optimization aimed at decreasing the threshold current density. We modified the reference EEL by employing additional low-refractive-index materials (plasmonic GaN, AlInN), to increase confinement of the optical modes and, in turn, further lower the threshold current density.

### 3.3. EEL ITO/GaN^++^

In this section, we analyze the impact of incorporating heavily doped GaN:Ge (plasmonic) layers as part of the n-cladding layer on the threshold current densities of the modeled device.

**Figure 6:**
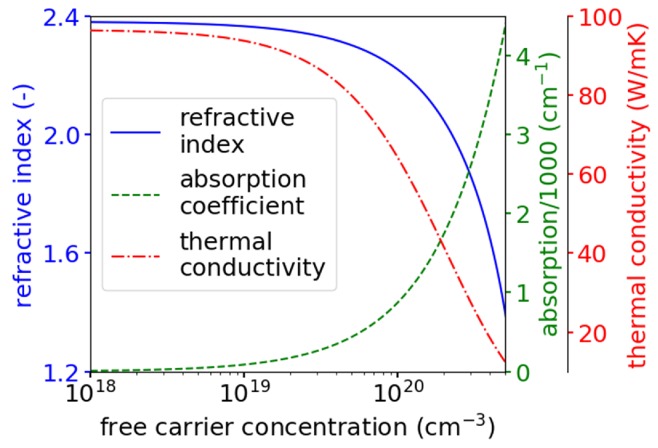
Refractive index, absorption coefficient and thermal conductivity as a function of free carrier concentration for heavily doped n-GaN.

Figure 6 presents the thermal and optical parameters used in the simulations as a function of the free carrier concentration of the plasmonic GaN. The refractive index and absorption coefficient were calculated using the Drude formula [12]. In our model, an increase of more than 10^19^ cm^−3^ in the free carrier concentration of the plasmonic GaN results in a considerable increase in the absorption coefficient and a decrease in thermal conductivity. According to the Drude model, in order to reduce the refractive index of GaN by 0.2, the concentration of free carriers must be 10^20^ cm^−3^.

**Figure 7:**
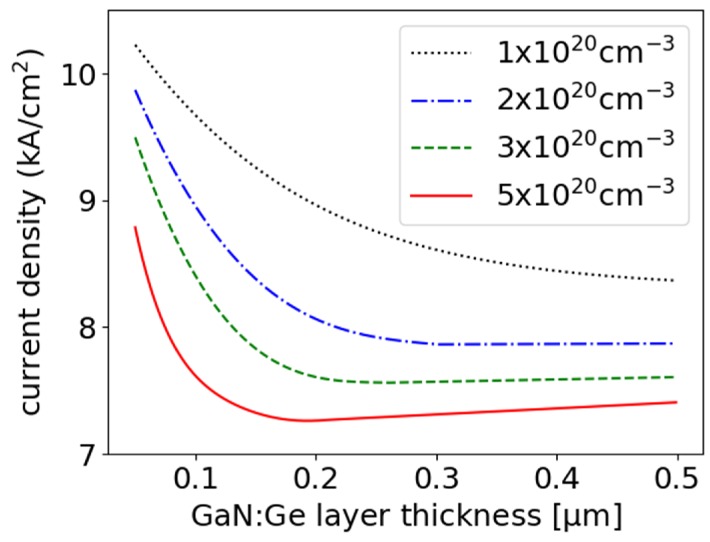
Threshold current densities calculated for the modeled EELs with plasmonic GaN as a function of the thickness of the plasmonic layer and free carrier concentrations.

The plasmonic GaN layers were separated from the InGaN n-waveguides using an n-GaN spacer. Our results refer to EELs with optimized structures with respect to the lowest possible threshold current densities. Such optimization of EEL with plasmonic GaN of fixed thickness and free carrier concentration requires estimation of the ITO and n-GaN spacer thicknesses. Figure 7 shows the threshold current densities calculated for EELs with plasmonic GaN layers of thicknesses and free carrier concentrations in the ranges of 0.1–0.5 μm and 1–5 × 10^20^ cm^−3^, respectively. The maximum in the latter range is based on data reported in [23]. An increase in the free carrier concentration of the plasmonic layer resulted in a decrease in both its optimal thickness and that of the n-GaN spacer, while the optimal ITO thickness increased (Table 4). Increasing the thickness of the plasmonic layer and the concentration of free carriers also resulted in a higher threshold current density. This effect was stronger for layers with higher free carrier concentrations.

**Figure 8:**
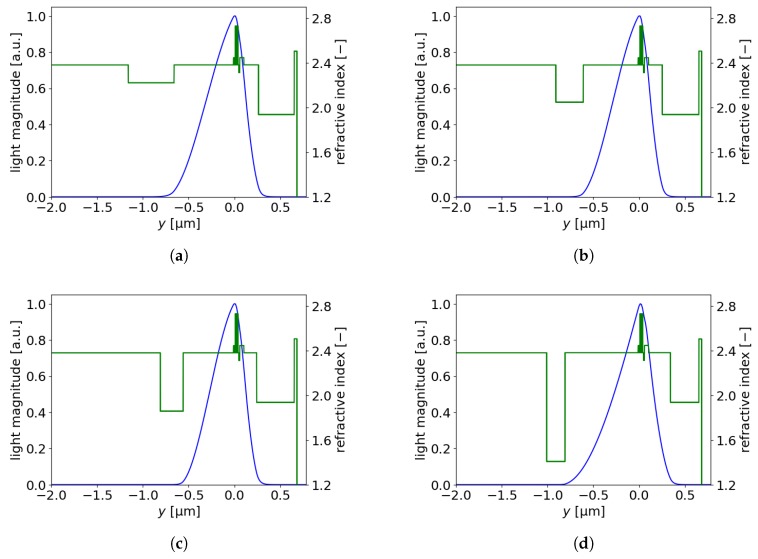
Fundamental mode and refractive index distributions along the *y*-axis for EELs with plasmonic n-claddings characterized by different free carrier concentrations/thicknesses: (**a**) $1\times 10^{20}$ cm^−3^/0.5 μm, (**b**) $2\times 10^{20}$ cm^−3^/0.3 μm, (**c**) $3\times 10^{20}$ cm^−3^/0.25 μm, (**d**) $5\times 10^{20}$ cm^−3^/0.2 μm.

Table 4 presents optimal designs for different free carrier concentrations in the plasmonic layer. The fundamental mode distributions and refractive indices along they-axis are presented in Figure 8a–d.

### 3.4. EEL ITO/AlInN

In comparison to n-GaN, n-AlInN has a lower refractive index (2.22 at 540 nm), higher absorption (23 cm^−1^ at 540 nm) and considerably lower thermal conductivity (5 W/(m·K)). In spite of its inferior thermal properties and absorption, the significant difference between the refractive indices of AlInN and GaN is expected to improve confinement of the optical mode in green EELs, and thereby contribute to lower the threshold current. We incorporated AlInN layers in the modeled EELs as part of their GaN/AlInN n-claddings.

**Figure 9:**
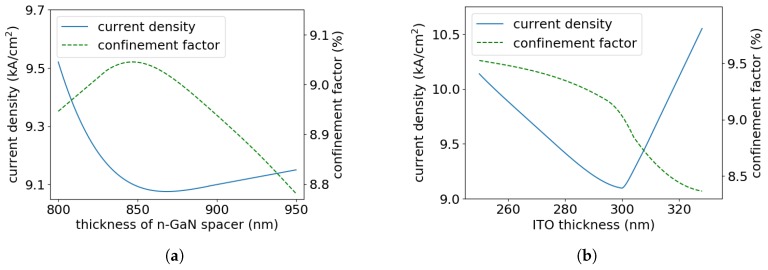
Threshold current densities and confinement factors calculated for EELs with 0.1-μm-thick n-AlInN layers (**a**) as a function of the thickness of the n-GaN spacer (ITO thickness set at 0.3 μm), and (**b**) as a function of ITO thickness (n-GaN spacer thickness set at 0.85 μm).

To obtain the lowest threshold current density, the positions and thicknesses of the AlInN layer in the n-side and of the ITO layer in the p-side were optimized simultaneously. The optimal AlInN layer position was achieved by modifying the thickness of the n-GaN spacer between the n-AlInN layer and the InGaN n-waveguide. The method employed to find the optimal ITO layer position was described in Section 3.2. Figure 9a,b show the current density and confinement factor as functions of the position and thickness of the ITO layer in a EEL with a 0.1-μm-thick AlInN layer separated from the n-waveguide by a 0.85-μm-thick n-GaN spacer. The minimum threshold current density was 9.10 kA/cm^−2^, which is 20% lower compared to the reference EEL. At the same time, the confinement factor of the active region was 2% larger than that in the reference case. The above results were found for an EEL with a 0.3-μm-thick ITO separated from the p-waveguide by a 0.25-μm-thick p-GaN.

**Figure 10:**
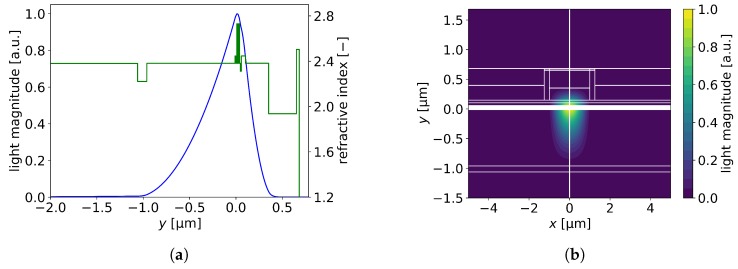
(**a**) Light magnitude and refractive index along the *y*-axis, (**b**) light magnitude in the xy-plane for an EEL with a 0.3-μm-thick ITO and 0.1-μm-thick n-AlInN layers.

Due to the significant asymmetry in the refractive index distribution along the y axis on either side of the active region (Figure 10a), modification of the thickness of any material on the p side requires appropriate modification of the layer thickness on the n side, to ensure the maximal confinement factor and hence minimal threshold current density. For instance, reducing the p-GaN thickness by 10 nm (followed by a 10-nm-increase in ITO) requires a corresponding reduction in the thickness of the n-GaN spacer by 50 nm to maintain low threshold current density.

With a thicker n-AlInN cladding layer, the GaN spacer can be thinned considerably. If the thickness of the ITO in the p-cladding is also increased (and the p-GaN thickness is decreased), this can improve optical mode confinement (Table 5). Optimization results for EELs with n-AlInN layers are presented in Table 5.

**Figure 11:**
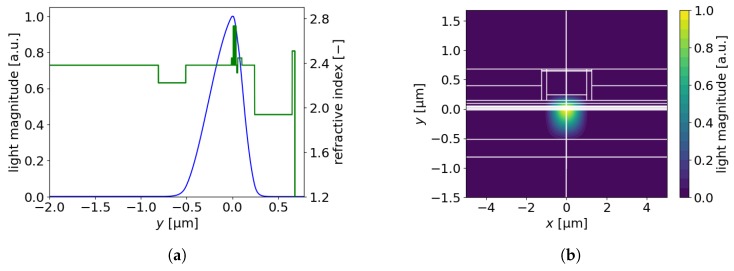
(**a**) Light magnitude and refractive index along the *y*-axis, (**b**) light magnitude in the xy-plane for an EEL with a 0.41-μm-thick ITO and 0.3-μm-thick n-AlInN layers.

**Figure 12:**
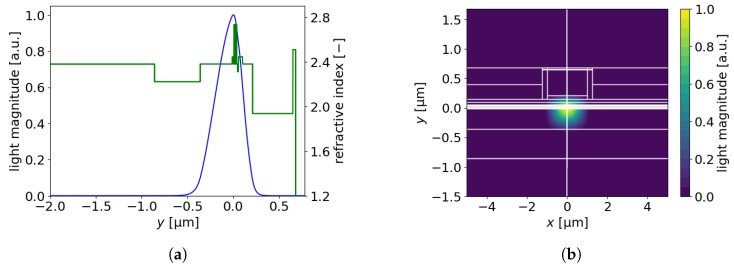
(**a**) Light magnitude and refractive index along the *y*-axis, (**b**) light magnitude in the xy-plane for an EEL with a 0.44-μm-thick ITO and 0.5-μm-thick n-AlInN layers.

Recently, AlInN layers with thicknesses of up to approximately 0.3 μm have been reported [24]. Figure 11a,b together with Table 5 present our results for EELs with n-AlInN cladding. If we compare the results for an EEL with 0.3-μm-thick n-AlInN to those in the reference case, the threshold current density decreased by 36%. In the case of an EEL with 0.5-μm-thick AlInN (see: Table 5), the threshold current decreased by 41% in comparison to the reference EEL. The EELs with thicker n-AlInN showed higher confinement factors (Figure 12a,b). In the case of an EEL with 0.5-μm-thick n-AlInN, the confinement factor was double that of the reference EEL.

## 4. Conclusions

In this paper, we investigated various construction designs for the green EEL in order to reduce the threshold current density. We started our research from the analysis of the heat-spreading properties of top gold metalization. It was found that a thicker p-Au contact leads to a significant decrease in the threshold current density. Due to the fact that for thicknesses above 1 μm the effects becomes less efficient, we decided to use the above value in the next stages of our study, where we considered: ITO as a partial replacement for the p-GaN in p-cladding, highly-doped plasmonic n-GaN layer, and lattice-matched n-AlInN as a part of the n-cladding or substrate.

ITO layers are commonly used in light emitting diodes, edge-emitting lasers and vertical-cavity surface-emitting lasers, and this technology seems to be already on a commercial level. Calculations performed for various ITO layer thicknesses showed that for a EEL with the p-cladding composed of 0.28-μm-thick ITO and 0.27-μm-thick p-GaN layers it is possible to obtain threshold current density of 11.3 kA/cm^2^. Using plasmonic n-GaN beneath the active region allows for a further reduction in the threshold current density. Its lowest value of 7.26 kA/cm^2^, was found for the plasmonic n-GaN layer with the thickness of 0.20 μm and the concentration of free charges of $5\times 10^{20}$ cm^−3^. However, in the plasmonic GaN, the strain increases with the increasing doping concentration. Although for $5\times 10^{20}$ cm^−3^ the compressive strain is as low as 0.26% [23], the thickness of these layers is as large as 0.20 μm, and when no tensile strained layers are used that could compensate that strain, the lattice defects (not considered in our calculations) are generated, which leads to the degradation of laser characteristics. These problems do not arise, when n-AlInN layer is introduced in the n-cladding, due to the fact that for indium content of 17% this material is lattice-matched to GaN. Incorporating n-AlInN, enables obtaining even lower values of a threshold current density than the use of plasmonic GaN. For n-AlInN of thickness of 0.5 μm the threshold operation of modeled EEL was obtained with threshold current density as low as 6.69 kA/cm^2^, which is about 40% reduction in comparison to the analogous value for the reference structure. However, ternary materials like AlInN have a higher thermal resistance than the binary ones. The thermal conductivity for Al_0.83_In_0.17_N is as low as 5 W/mK, which affects the thermal properties of the structure. Although in the case of threshold operation this effect is not so significant, for the over-threshold operation of the laser, it can be a noticeable drawback.

The most promising solution when the laser operates near the threshold is n-AlInN layers as cladding that enables high mode confinement. When laser operates significantly above the threshold, thermal management is dominating factor limiting laser efficiency, then the plasmonic GaN layer of moderate doping level close to $1\times 10^{20}$ cm^−3^ should be considered as cladding layer due to its good thermal conductivity.

## Figures and Tables

**Table 1:** Details of the green EEL. EBL—electron blocking layer, MQW—multi quantum well.

layer	Material	Thickness [μm]
p-contact	Ti/Au	0.03/1
isolation	SiO_2_	0.25
p-cladding	ITO/p-GaN	0.28/0.27
p-waveguide	p-In${}_{0.08}$Ga${}_{0.92}$N	0.045
EBL	p-Al${}_{0.2}$Ga${}_{0.8}$N	0.010
active region	MQW In${}_{0.29}$Ga${}_{0.71}$N/GaN	0.0027/0.01
n-waveguide	n-In${}_{0.08}$Ga${}_{0.92}$N	0.010
n-cladding	n-GaN	0.55
substrate	n-GaN	50
n-contact	Al/Au	0.03/0.3

**Table 2:** Thermal and electrical parameters of materials used in simulations. $\kappa_{300\;K}$ and $\kappa_{400\;K}$—thermal conductivity at 300 K and 400 K, $\sigma_{300\;K}$ and $\sigma_{400\;K}$—electrical conductivity at 300 K and 400 K.

Material	Doping	Thickness	$\kappa_{300\;K}$	$\kappa_{400\;K}$	$\sigma_{300\;K}$	$\sigma_{400\;K}$
	[cm^−3^]	[μm]	[W/(m·K)]	[W/(m·K)]	[S/m]	[S/m]
p-GaN	Mg: $2\times 10^{19}$	0.21–0.37	92	61	95	309
p-In${}_{0.08}$Ga${}_{0.92}$N	Mg: $1\times 10^{19}$	0.045	23	20	284	280
p-Al${}_{0.2}$Ga${}_{0.8}$N	Mg: $5\times 10^{19}$	0.010	13	12	22	70
n-GaN (substrate)	Si: $2\times 10^{18}$	50	166	110	$7.9\times 10^{3}$	$7.4\times 10^{3}$
n-In${}_{0.08}$Ga${}_{0.92}$N	Si: $5\times 10^{18}$	0.01	28	26	636	703
n-Al${}_{0.83}$In${}_{0.17}$N	Si-doped	0.1–0.5	5	5	$2.4\times 10^{4}$	$2.4\times 10^{4}$
n-GaN (plasmonic)	Ge: $1\times 10^{20}$	0.05–0.50	54	36	$2\times 10^{5}$	$2\times 10^{5}$
n-GaN (plasmonic)	Ge: $2\times 10^{20}$	0.05–0.50	35	24	$3\times 10^{5}$	$3\times 10^{5}$
n-GaN (plasmonic)	Ge: $3\times 10^{20}$	0.05–0.50	23	16	$4\times 10^{5}$	$4\times 10^{5}$
n-GaN (plasmonic)	Ge: $5\times 10^{20}$	0.05–0.50	10	7	$5\times 10^{5}$	$5\times 10^{5}$
GaN (barrier)	undoped	0.010	61	41	255	242
n-In${}_{0.29}$Ga${}_{0.71}$N (QW)	Si-doped	0.0027	2	2	250	278
SiO_2_	n/a	0.25	1	1	$1\times 10^{-13}$	$1\times 10^{-13}$
ITO	n/a	0.18–0.44	3	3	$1\times 10^{6}$	$1\times 10^{6}$
Au	n/a	0.1–3.0	316	311	$4.4\times 10^{7}$	$3.2\times 10^{7}$
Al	n/a	0.03	180	180	$3.8\times 10^{7}$	$3.8\times 10^{7}$
Ti	n/a	0.03	22	20	$2.2\times 10^{6}$	$1.6\times 10^{6}$
PbSn	n/a	1	50	50	$6\times 10^{6}$	$6\times 10^{6}$
Cu	n/a	5000	403	393	$5.8\times 10^{7}$	$4.2\times 10^{7}$

**Table 3:** Optical parameters of materials used in simulations. $n_{r,300\;K}$ and $n_{r,400\;K}$—refractive index at 300 K and 400 K, $\alpha_{300\;K}$ and $\alpha_{400\;K}$—absorption coefficient at 300 K and 400 K.

Material	$n_{r,300\;K}$	$n_{r,400\;K}$	$\alpha_{300\;K}$ [1/cm]	$\alpha_{400\;K}$ [1/cm]
p-GaN	2.3787	2.3881	100	100
p-In${}_{0.08}$Ga${}_{0.92}$N	2.4429	2.4530	100	100
p-Al${}_{0.2}$Ga${}_{0.8}$N	2.3099	2.3181	100	100
n-GaN	2.3787	2.3881	10	10
p-In${}_{0.08}$Ga${}_{0.92}$N	2.4429	2.4530	10	10
n-Al${}_{0.83}$In${}_{0.17}$N	2.2202	2.2260	23	23
n-GaN (Ge: $1\times 10^{20}$)	2.2179	2.2267	870	870
n-GaN (Ge: $2\times 10^{20}$)	2.0445	2.0526	1745	1745
n-GaN (Ge: $3\times 10^{20}$)	1.8550	1.8623	2615	2615
n-GaN (Ge: $5\times 10^{20}$)	1.4009	1.4065	4350	4350
n-In${}_{0.29}$Ga${}_{0.71}$N (QW)	2.7310	2.7431	gain	gain
SiO${}_{2}$	1.4795	1.4795	0	0
ITO	1.9357	1.9357	1920	1920
Au	0.3710	0.3710	$4.9\times 10^{5}$	$4.9\times 10^{5}$
Ti	2.5081	2.5081	$8.0\times 10^{5}$	$8.0\times 10^{5}$

**Table 4:** Optimization results for EELs with ITO/GaN p-claddings and GaN/plasmonic-GaN n-claddings. $n_{\mathrm{plasm}}$—free carrier concentration in the GaN:Ge plasmonic layer, $t_{\mathrm{plasm}}$—thickness of the GaN:Ge plasmonic layer, $t_{\mathrm{GaN}}$—n-GaN spacer thickness, $t_{\mathrm{ITO}}$—ITO layer thickness, $j_{\mathrm{th}}$—threshold current density, Γ—confinement factor.

$n_{\mathrm{plasm}}$	$t_{\mathrm{plasm}}$	$t_{\mathrm{GaN}}$	$t_{\mathrm{ITO}}$	$j_{\mathrm{th}}$	Γ
[cm^−3^]	[μm]	[μm]	[μm]	[kA/cm^2^]	[%]
1 × 10${}^{20}$	0.50	0.65	0.39	8.39	9.8
2 × 10${}^{20}$	0.30	0.60	0.40	7.87	10.5
3 × 10${}^{20}$	0.25	0.55	0.41	7.56	11.2
5 × 10${}^{20}$	0.20	0.50	0.42	7.26	12.1

**Table 5:** Optimization results for EELs with ITO/GaN p-claddings and GaN/AlInN n-claddings. $t_{\mathrm{AlInN}}$—n-AlInN layer thickness, $t_{\mathrm{GaN}}$—n-GaN spacer thickness, $t_{\mathrm{ITO}}$—ITO layer thickness, $j_{\mathrm{th}}$—threshold current density, Γ—confinement factor.

$t_{\mathrm{AlInN}}$	$t_{\mathrm{GaN}}$	$t_{\mathrm{ITO}}$	$j_{\mathrm{th}}$	Γ
[μm]	[μm]	[μm]	[kA/cm^2^]	[%]
0.1	0.85	0.30	9.10	9.0
0.3	0.50	0.41	7.26	11.2
0.5	0.35	0.44	6.69	13.6
